# Changes in Dendritic Spine Morphology and Density of Granule Cells in the Olfactory Bulb of *Anguilla anguilla* (L., 1758): A Possible Way to Understand Orientation and Migratory Behavior

**DOI:** 10.3390/biology11081244

**Published:** 2022-08-21

**Authors:** Riccardo Porceddu, Cinzia Podda, Giovanna Mulas, Francesco Palmas, Luca Picci, Claudia Scano, Saturnino Spiga, Andrea Sabatini

**Affiliations:** 1 Department of Life and Environmental Sciences (DiSVA), University of Cagliari, Via T. Fiorelli 1, 09126 Cagliari, Italy; 2 Consorzio Nazionale Interuniversitario per le Scienze Mare (CoNISMa), Piazzale Flaminio 9, 00196 Roma, Italy

**Keywords:** catadromous fish olfaction, olfactory bulb, olfactory granule cell, dendritic spine development, European eel, orientation, migratory behaviour

## Abstract

**Simple Summary:**

The olfactory bulb can process odour cues through granular cells (GCs) and dendritic spines, changing their synaptic plasticity properties and their morphology. The GCs’ dendritic spines density and morphology were analysed in *Anguilla anguilla,* considering the olfaction as a driver involved in fish orientation and migration. For the head and neck morphology, spines were classified as mushroom, long thin, stubby, and filopodia. Spines’ density decreased from juvenile migrants to no-migrant stages and increased in the adult migrants. Spines’ density was comparable between glass and silver eels as an adaptation to migration, while at non-migrating phases, spines’ density decreased. For its phylogenetic Elopomorph attribution and its complex life cycle, *A. anguilla* could be recommended as a model species to study the development of dendritic spines in GCs of the olfactory bulb. Considering the role of olfaction in the orientation and migration of *A. anguilla*, the modification of environmental stimuli (ocean alterations and climate change) could represent contributing factors that threaten this critically endangered species.

**Abstract:**

Olfaction could represent a pivotal process involved in fish orientation and migration. The olfactory bulb can manage olfactive signals at the granular cell (GC) and dendritic spine levels for their synaptic plasticity properties and changing their morphology and structural stability after environmental odour cues. The GCs’ dendritic spine density and morphology were analysed across the life stages of the catadromous *Anguilla anguilla*. According to the head and neck morphology, spines were classified as mushroom (M), long thin (LT), stubby (S), and filopodia (F). Total spines’ density decreased from juvenile migrants to no-migrant stages, to increase again in the adult migrant stage. Mean spines’ density was comparable between glass and silver eels as an adaptation to migration. At non-migrating phases, spines’ density decreased for M and LT, while M, LT, and S density increased in silver eels. A great dendritic spine development was found in the two migratory phases, regressing in trophic phases, but that could be recreated in adults, tracing the migratory memory of the routes travelled in juvenile phases. For its phylogenetic Elopomorph attribution and its complex life cycle, *A. anguilla* could be recommended as a model species to study the development of dendritic spines in GCs of the olfactory bulb as an index of synaptic plasticity involved in the modulation of olfactory stimuli. If olfaction is involved in the orientation and migration of *A. anguilla* and if eels possess a memory, these processes could be influenced by the modification of environmental stimuli (ocean alterations and rapid climate change) contributing to threatening this critically endangered species.

## 1. Introduction

Olfaction is a pivotal process involved in many behaviours of fish (orientation, migration, feeding, defence, spawning, and schooling) whose life is entirely restricted to the aqueous environment [1,2,3]. The behavioural significance of the signals arriving at the brain through different channels varies greatly from one species to another. The importance of olfaction in the behaviour pattern known as homing is displayed by many fish species (e.g., eel, rainbow trout, Pacific, and Atlantic salmon) [4,5,6,7,8,9,10].

Olfaction cues, reached first by olfactory neurons, are managed by the olfactory bulb (OB), whose projections lead to higher telencephalic areas [11,12] and reciprocally receive projections from the telencephalon itself [12,13,14]. Different neurobiological studies have been performed on fish olfactory circuits, searching for a possible area involved in olfactory memory formation. For instance, in *Cyprinus carpio* (L., 1758), the OB shows general properties for neural plasticity in vivo and in vitro as examples of input-specific, activity-dependent synaptic plasticity [15,16,17]. These synaptic activities can take place at the dendro-dendritic synapses between glutamatergic mitral cells (MCs) and granular cells (GCs, interneurons Gamma Amino Butirric Acid GABA releasing) at the peripheral dendrites. Their activity is also regulated by synapses with noradrenergic afferent projections by the telencephalon, which are mainly located at the deep dendrites [16,17]. Furthermore, OB shows that both intrinsic and environmental factors can rearrange the dendritic spines of GCs [18,19,20,21,22,23,24]. A single dendritic spine of a GC can change its morphology and structural stability after environmental odour enrichment or removal [25]. Dendritic spines are the most common postsynaptic structures of most excitatory synapses, connecting presynaptic and postsynaptic neurons [26]. They consist of a small bulbous head connected to its dendrite through a neck, which provides a biochemical and electrochemical compartmentalisation of the synapse. The dimension of the head is directly proportional to the quantity of structural synaptic proteins and postsynaptic functional receptors [27]. Moreover, synaptic activity is associated with physical growth or shrinkage of the spine [28].

Dendritic spines can mutate in various shapes and sizes, depending on brain areas, cell types, and animal species [29]. Their structure is classified into four conventional classes according to the morphological features of the head and neck: mushroom, long thin, stubby, and filopodia [25,30,31]. Mushroom spines are characterised by a large head and a small neck, able to establish strong synaptic connections, showing the longest lifetime and constituting the sites of long-term memory storage [32,33]. Long thin spines are structurally similar to mushroom spines but have a smaller head. They are more changeable and, for this reason, can be considered the learning spines, able to learn new memories during synaptic plasticity, followed by head growth [32,33]. Stubby spines usually do not have a neck and are the predominant spine type during early postnatal development stages, but a small amount is present also in adulthood due to the disappearance of mushroom spines [34]. Filopodia are long dendritic protrusions without a well-defined head, typically observed in young developing neurons [35]. These structures are mobile and flexible but with a short lifetime [36].

The shape, structural organisation, synaptic function, and morphological rearrangements of dendritic spines are potentially related to development or experience [19,20,21,22,37,38,39].

Furthermore, the stability of spines determines the maturity of neural circuits and their maturation is related to learning, memory formation and storage, and memory consolidation [25,33,37,40,41,42,43].

The catadromous European eel *Anguilla anguilla* (L., 1758) during its life undertakes one of the most extraordinary migrations in the animal kingdom [44]. Indeed, the European eel crosses the entire Atlantic Ocean twice, first as larvae and finally as an adult. The species spawns in the Sargasso Sea, then leptocephali larvae migrate towards the European and North African coasts for more than 5000 km [45,46], transported by the Gulf Stream [47,48,49,50]. Larvae metamorphose into unpigmented glass eels [51] able to reach continental areas [47,52] where, under stimulation by chemical attractants (pheromones, green odors, amino acids, and bile salts), magnetic and lunar orientation mechanisms, and/or salinity gradients [53,54,55,56,57,58,59,60] recruit estuarine environments, starting their upstream migration [61]. Reaching continental waters, glass eels pigment into juvenile yellow eels (elvers), developing all morphological and physiological features necessary for life in inland waters [62,63]. Eels spend most of their lifetime in these habitats (5–25 years or more) at the adult yellow eel stage, then metamorphose into silver eels towards sexual maturity during seaward migration [47,64,65,66,67]. At this stage, eels swim across the Atlantic Ocean to the spawning area in the Sargasso Sea to die after spawning [45,66,67].

**Figure 1:**
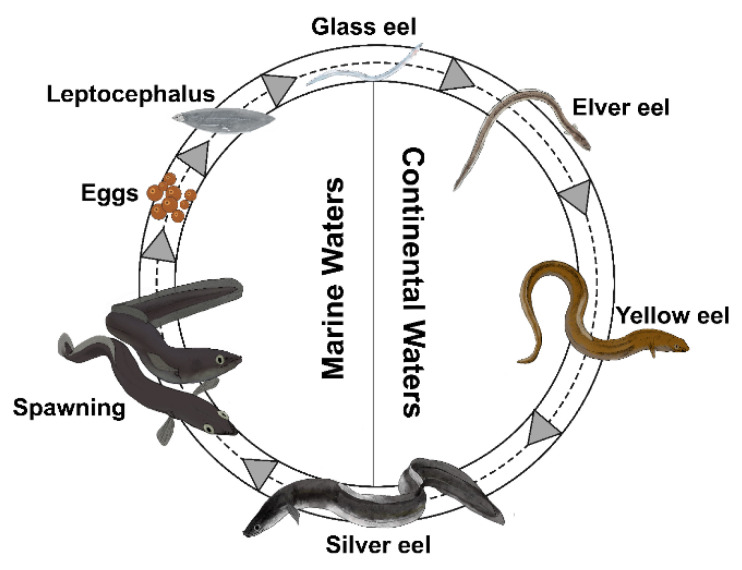
The life cycle of the European eel (*Anguilla anguilla*).

Because of the complex life cycle (Figure 1), characterized by metamorphosis, a migratory behaviour, and the capacity to live in different habitats (marine, brackish, and freshwater) [44], the European eel developed one of the most sensitive olfactory systems among fish and olfaction plays a central role in its life [44,68,69,70,71]. However, the morphological development of brain areas involved in olfaction remains unknown.

To fill these gaps, in general, this study aimed to investigate the development of dendritic spines in the secondary dendritic trunk of OB GCs of the European eel, which resulted as the peripheral ones, known for the presence of synapses between MCs and GCs, characterised by general properties for neural plasticity [15,16,17]. In particular, this study aimed: (1) to investigate the morphological characterisation of dendritic spines along the entire eel’s life cycle and (2) using a cytomorphological Golgi staining approach, the differences in the density of these structures for each eel’s continental life stage.

## 2. Materials and Methods

### 2.1. Eel Samples

Eels were collected between November 2019 and January 2021 from the Pramaera River (Central-Eastern Sardinia, Italy). The Pramaera River is a typical Mediterranean small watercourse characterised by biseasonal climatic features, with hot arid summers, rainy autumn/winter seasons along with extreme precipitation events, determined irregular flow, and strong seasonal hydrological fluctuations [72,73,74,75]. The river showed well-oxygenated waters (dissolved oxygen = 9.40 ± 12.35 mg L^−1^), good conductivity (1324.16 ± 1564.71 µS cm^−1^), and typically Mediterranean average water temperatures (16.01 ± 5.32 °C). Erosion was not very evident indicating a high level of naturalness and integrity and a good fish suitability [76]. Euryhaline fish species (e.g., mullets and seabass), and mostly European eels populate this river [77]. The extension of the watercourse is 10 km, with a catchment area of 180.7 km^2^, currently devoid of fluvial interruptions of anthropogenic origin (i.e., dams or other anthropogenic barriers).

Nineteen animals were used for the experiment. According to the peak migration periods of this species in Sardinia [77], five glass eels and five elvers were caught using experimental fyke nets. Five yellow and four male silver eels were captured using low-frequency, pulsed DC electrofishing. All individuals were immediately stored in cool and aerated water and anaesthetised by immersion in a bath of MS 222 until the termination of opercular movements [78] and measured for total length (TL, cm) and total weight (TW, g). Then, animals were sacrificed in situ using decapitation, conforming with the guidelines and protocols approved by the European Community and Italian legislation for the protection of animals used for scientific purposes (Directive 2010/63/UE L 276 20/10/2010, implemented by Italian Legislative Decree 26/2014). Finally, eel heads were immediately fixed in 4.0% paraformaldehyde in phosphate buffer solution (PBS, pH 7.40) and stored on ice for transfer to the laboratory for subsequent analysis.

### 2.2. Golgi-Cox Processing

In the laboratory, the whole brain (including rostral and caudal portions) was removed from the skull and kept in paraformaldehyde solution at 4 °C overnight. All brains were washed in PBS, weighed (brain weight, BW), and placed in 20 mL Golgi-Cox solution (known for randomly providing the most complete morphology about the 5% of the total neuron population) [79,80] for 2 weeks at room temperature in the dark. Brains were quickly washed in distilled water and transferred in a 30% sucrose plus 0.2% Sodium Azyde solution in PBS for cryoprotection for a minimum of 3 days at 4 °C to accurately remove the Golgi-Cox solution in excess [81].

**Figure 2:**
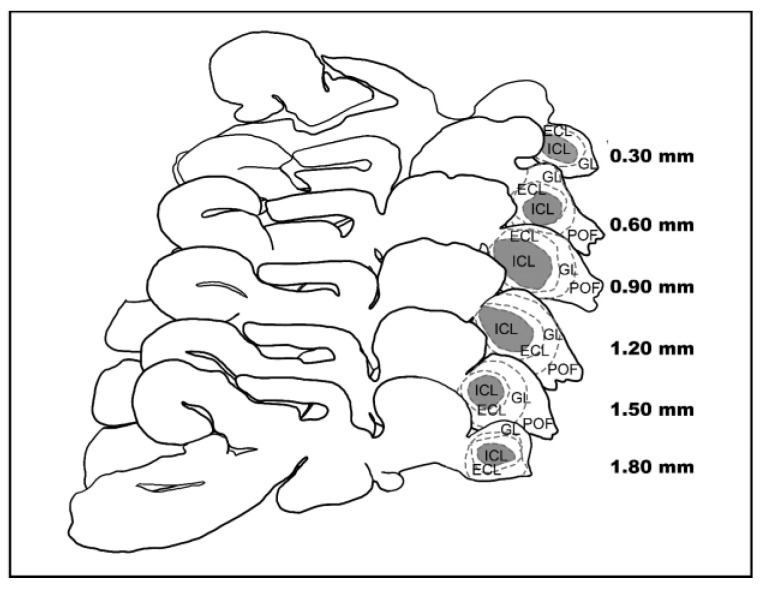
Sagittal slices of the eel’s brain. Representation of the sagittal slices of a male Silver eel’s brain. The ICL (grey area) of the left OB is included.

Afterward, brains were included in 35% gelatine/25% sucrose in PBS and cut at 80 to 100 µm thick sagittal slices using a vibratome (Leica VT1000S). Slices were collected in a cryoprotectant in series, selecting those involved in OB, using a stereomicroscope to identify the target brain area according to the zebrafish brain atlas ‘Neuroanatomy of the Zebrafish Brain’ [82] as a reference (Figure 2). Slices were developed using the procedure described by Kolb and McClimans [83], dehydrated, cleared, and mounted with Canada balsam.

### 2.3. Laser Scanning Confocal Microscopy

**Figure 3:**
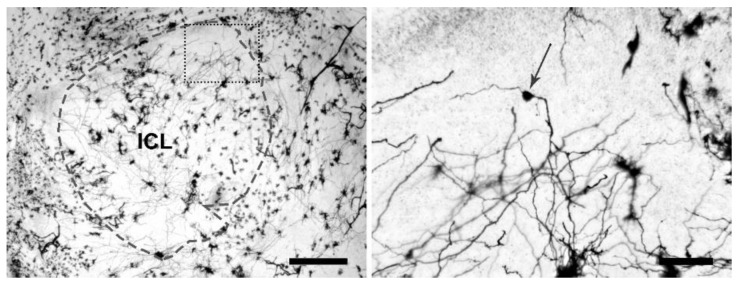
Golgi-Cox Staining in Light Microscopy. Left image shows the ICL of a male Silver eel’s OB (grey dotted line) (scale bar = 250 µm). Right image shows a magnification of the rectangular field in the left image (black dotted line), where a subtype IIIb GC (somata indicated by the grey arrow) is located (scale bar = 50 µm).

Quantitative analysis was performed using a Leica 4D confocal laser scanning microscope (CLSM) with an argon-krypton laser (Leica, Heidelberg, Germany). Confocal images were generated using 100× oil (n.a. = 1.3) in reflection mode (488 excitation wavelength). Each frame (512 lines and 512 columns) was acquired eight times and averaged to obtain noise-free images. Confocal images were obtained from the maximum number of scans allowed by the dendrite thickness. Optical sections, usually at consecutive intervals of 0.5 μm, were imaged through the depth of the labelled neurons and saved as image stacks. All confocal images were white labelled on a black background in grayscale ranging from 0 (black) to 255 (white) and processed in grayscale values with Scanware 4.2a Leica. Criteria for morphological analyses were: (i) internal cell layer, where GABAergic GCs were more concentrated, with somata diameter of 7 to 10 μm [84] were considered; (ii) only clearly and completely countable GCs were classified; (iii) type IIIb sub-cells [85] were included (Figure 3).

### 2.4. Rendering

**Figure 4:**
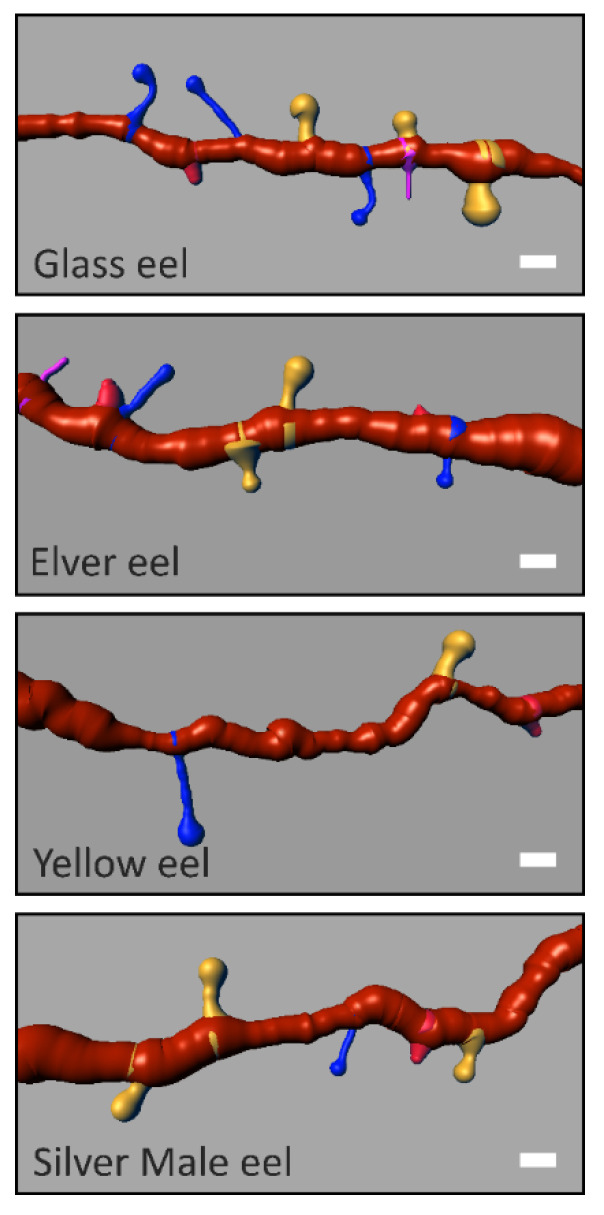
Representative GC’s secondary dendritic branches (orange). Spine class densities and differences among the Glass, the Elver, the Yellow and Silver male eels. (Scale Bar = 1 µm). Mushroom spine (yellow); long thin spine (blue); stubby spine (red); filopodia (magenta).

Image analysis was performed using the software Bitplane Imaris 7.4.2. by two independent observers blind to the eel life stage using the libraries Filament Tracer and Classifying Spine. Spine density was calculated by tracing at least a 10 μm long spline curve along the secondary dendritic trunk of GCs (Figure 4).

Primary dendritic trunks, known for being primarily innervated by inputs of telencephalic origin [16,17] were not considered. For each eel continental life stage, for spine density evaluation, about 75 dendritic segments were generated. According to the head and neck morphological/metric criteria reported by Spiga et al. [30], spines were classified into four classes: stubby spines (S) (no distinguishable head and total length less than 1 µm), mushroom spines (M) (head diameter greater than the maximum diameter of the neck, well-formed head, and neck diameter greater than its length), long thin spines (LT) (head diameter greater than the maximum diameter of the neck, well-formed head, and neck length greater than its diameter), and filopodia (F) (no distinguishable head and total length greater than 10 µm).

### 2.5. Statistical Analysis

The relationships between TL, TW, BW, and spine density in the OB GC secondary dendritic trunk were evaluated for each specimen using linear correlation analysis (correlation coefficient R^2^). Before linear regression, the extreme difference between the values of body characteristics was down-weighted by applying a log-transformation. Spine density was checked for normality (Shapiro–Wilk’s test, S–W, *p* < 0.05). Therefore, differences in spine density among life stages and spine classes were analysed by the nonparametric Kruskal-Wallis test (K-W test) to verify the equality of the medians between different groups. When significant differences were obtained, pairwise comparisons were conducted using Dunn’s post hoc test (Z test). All values were expressed as the mean and standard error (±SE) unless otherwise indicated. Significance was set at *p* < 0.05. All data were analysed by R [86].

## 3. Results

Basic statistics relative to TL, TW, and BW of different eel life stages are reported in Table 1.

From the image analysis, 308 segments (about 10 μm long) from secondary dendritic trunks in eel OB GCs were collected and dendritic spine classes were characterised (Figure 4). 

**Figure 5:**
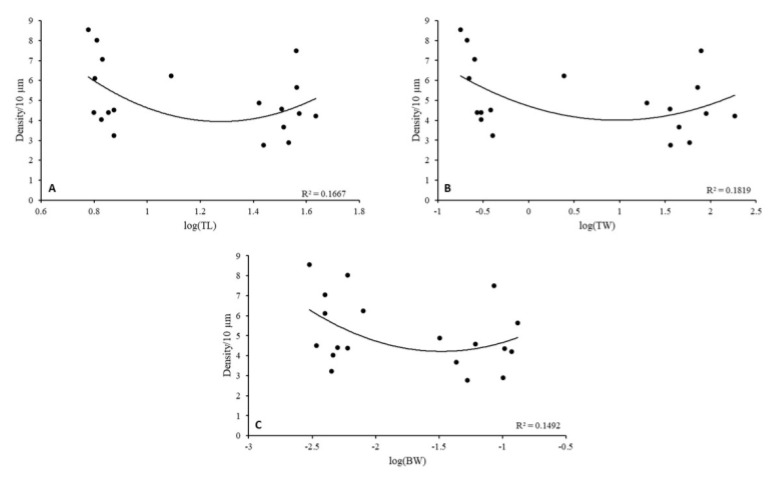
Relationships between eels’ body characteristics and spine total density from secondary dendritic trunks in the GCs of the OB. (**A**) log-transformed total length (TL), (**B**) log-transformed total weight (TW), (**C**) log-transformed brain weight (BW).

Linear regression between body characteristics (TL, TW, and BW) and spine density for each eel life stage showed no significant correlations (R^2^ coefficient < 0.7, *p* < 0.05). However, the highest total spine density was observed in relation to eels’ body characteristics for the glass eel stage. In elvers and yellow eels, the density decreases and increases again at the silver eel stage (Figure 5).

**Figure 6:**
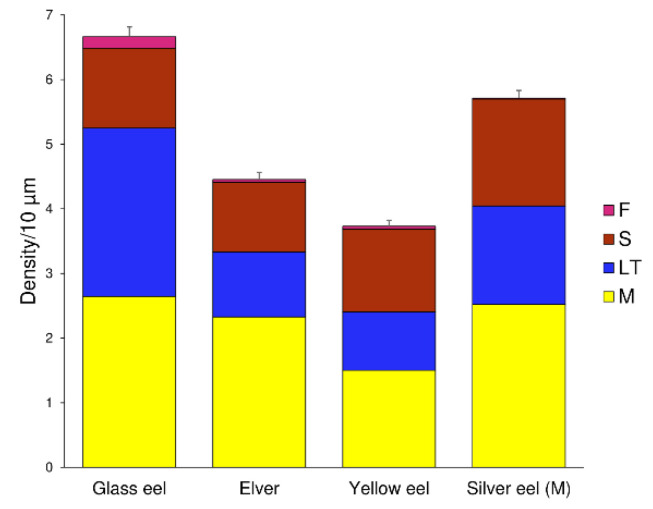
Dendritic spine density per 10 μm of dendritic trunk length ± SEM, grouped for eels’ life stage and for spine class. Bars are repartitioned according to the life stage of eels and the dendritic spine class, respectively (M: mushroom in yellow, LT: long thin in blue, S: stubby in red, F: filopodia in pink).

Total spine density was abundant in the glass eel stage (6.67 ± 0.15 spines/10 µm), reducing progressively in the elver (4.45 ± 0.11 spines/10 µm) and yellow eel (3.73 ± 0.09 spines/10 µm) stages, to increase again in the silver eel stage (5.71 ± 0.12 spines/10 µm) (Figure 6). Significant differences were highlighted in the median values of total spine density among life stages (K-W: 42.77, *p* < 0.0001). Post hoc Dunn’s test showed no statistical differences, only between elvers and yellow eels (Z: −1.42, *p* > 0.05).

Densities within dendritic spine classes (M, LT, S, F) revealed significant differences for each eel life stage (Table 2).

**Figure 7:**
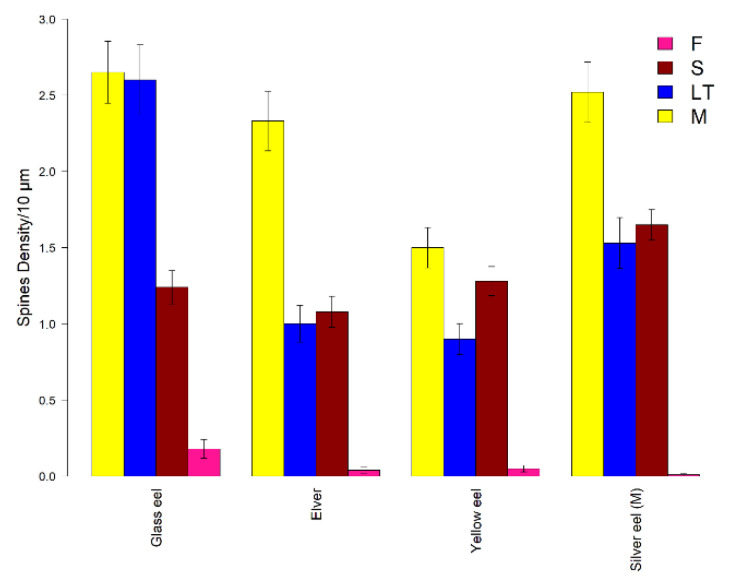
Mean density of dendritic spines. Mean spine density per 10 µm in the European eel per life stage and per spine class (M: mushroom in yellow, LT: long thin in blue, S: stubby in red, F: filopodia in pink). Error bars represent standard error of the mean.

For glass eels (K-W: 23.52, *p* < 0.05), greater mean densities were found for M and LT spine classes (2.65 ± 0.20 spines/10 µm, and 2.60 ± 0.23 spines/10 µm, respectively) that, indeed, were detected as statistically similar (Z: 0.48, *p* > 0.05) (Figure 7). For elvers (K-W: 130.19, *p* < 0.05), M spines were the most abundant class (2.33 ± 0.20 spines/10 µm). In addition, no differences were observed between S and LT spine classes (Z: 0.87, *p* > 0.05) (Figure 7). In the yellow eel stage (K-W: 47.23, *p* < 0.05), both M and S classes showed higher density values (1.50 ± 0.13 spines/10 µm, and 1.28 ± 0.10 spines/10 µm, respectively) with no statistical differences (Z: −0.51, *p* > 0.05) (Figure 7). Lastly, greater density spines (K-W: 7.90, *p* < 0.05) were obtained for the M spine class (2.52 ± 0.20 spines/10 µm) for silver eels. Furthermore, no differences were detected between S and LT classes (Z: 1.53, *p* > 0.05) (Figure 7).

Based on the analysis of the density of different dendritic spine classes, the total spine density was greater in M spines (2.25 ± 0.19 spines/10 µm), lower in F (0.07 ± 0.03 spines/10 µm), and intermediate in LT (1.51 ± 0.18 spines/10 µm) and S (1.31 ± 0.10 spines/10 µm) spines. Moreover, median spine density was significantly different among all spine classes (K-W: 571.23, *p* < 0.001). Post hoc Dunn’s test explained no statistical differences only between LT and S spine classes (Z: 0.52, *p* > 0.05).

Considering all dendritic spine classes separately (Figure 7), M and LT spines showed a reduction in spine density in the elver and yellow eels’ stages to increase again in the silver eel stage. Regarding the S spine class, a slight decrease in spine density was found between glass eel and elver stages to increase again in yellow and silver eels. Finally, the density of the F class decreased progressively until it almost disappeared in silver eels. Within each spine class, significant differences were identified (Table 3).

The density in M spines was quite similar between glass eels and elvers (Z: 1.17, *p* > 0.05), glass eels and silver eels (Z: −0.37, *p* > 0.05), and elvers and silver eels (Z: 0.81, *p* > 0.05), respectively, showing greater values (glass eel 2.65 ± 0.20 spines/10 µm; silver eel 2.52 ± 0.20 spines/10 µm; elver 2.33 ± 0.20 spines/10 µm). Regarding LT spine density, a greater value was detected in the glass eel stage (2.60 ± 0.23 spines/10 µm) and no differences were observed between the elver and yellow eel stages (Z: −0.37, *p* > 0.05). For S spines, the most abundant were in the silver eel stage (1.65 ± 0.10 spines/10 µm). The pairwise Dunn’s test confirmed that this stage was statistically different from the others (silver eels vs glass eels: Z: 3.01, *p* < 0.05, silver eels vs. elvers: Z: 3.98, *p* < 0.05, silver eels vs. yellow eels: Z: −2.45, *p* < 0.05). F class showed greater density in the glass eel life stage (0.18 ± 0.06 spines/10 µm), with the only significant difference between the glass eel and silver eel stages (Z: −2.70, *p* < 0.05).

## 4. Discussion

Although the role of olfactory stimuli in the modulation of different functions in fish is known (e.g., social relationships, prey or predator recognition, and the search for food) [87], some studies have suggested a key role of olfaction in migration in the genus *Anguilla* [68,69].

In this study, we tried to understand if the olfactory system could play a specific role in the migratory behaviour and orientation of *A. anguilla,* focusing on the development of dendritic spines in OB GCs as an index of synaptic plasticity involved in the possible modulation of olfactory stimuli that drive these behaviours.

Considering the specific evolution of dendritic spines, starting from the pattern described in the vertebrate central nervous system, it is well known that the total spine density decreases from younger to older individuals, mostly connected with ageing [25,88,89]. In the OB, mushroom and long thin spines are the most abundant spine classes, with mushroom spines representing a more stable synapse than long thin spines [25]. Mushroom spines are characterised by a large amount of structural synaptic proteins and postsynaptic receptors [22,90,91] and by long-term potentiation [92]. Furthermore, long thin morphology shows a smaller head and a lower number of synaptic proteins and postsynaptic receptors than the mushroom shape [22,90]. Stubby spines can represent immature spines [90,91,93,94] that may disappear or evolve into long thin spines [37]. During the second postnatal week of young mammals, dendritic filopodia can emerge and interact with other neurons to form nascent synapses [91,95], which can later develop into stubby spines [96,97,98].

In general, results showed that the total dendritic spine density decreases progressively from glass eels to non-migrant stages (elvers and yellow eels) to increase again in the adult migrant silver stage. The amount of dendritic spines is comparable between glass eels and silver eels, demonstrating a quantitative and qualitative adaptation of these structures during the two migratory phases of the species. It was hypothesised that this overall trend is initially dictated by the decrease in the density of mushroom and long thin spines during the non-migrating trophic phase. Subsequently, the total spine density increases again in the silver eel stage due to the increment of mushroom, long thin, and stubby spines. Therefore, this model is in contrast to what is known in other vertebrates, revealing a relationship between the trend of the total spine density and the catadromous life cycle and the consequent two migratory phases of this species rather than with the ageing of the animal.

By analysing the single spine classes, mature mushroom and long thin spines were very similar in the migratory glass eel stage. Instead, in adult silver eels, there was a similar increase between long thin and stubby spines. These results suggested that there would be an increase in synaptogenesis in juvenile and adult stages, correlated by the increment in the number of mushroom, long thin, and stubby spines, with respect to elver and yellow eel stages. Furthermore, in the silver eel stage, the increase in these three spine classes suggested that this migratory phase could stimulate the processing of a large amount of new olfactory cues, which may correspond to only reproductive functions, including pheromone detection [99]. Stubby spines would constitute the source of the future mature mushroom and long thin spines, necessary in the migration phase to reach the spawning area. Higher-density values of filopodia were found at the juvenile glass eel stage and this value gradually decreased in the subsequent development stages, which were statistically homogeneous with each other. Although it was the lowest abundant class, their greatest production in the glass eel stage could be attributable to the possible role of these spines in the neural network formation in the OB during the still-stabilising juvenile stage. However, these results were in line with those reported in the developmental model of filopodia in other vertebrates, where a greater filopodia production was observed in the first weeks of life [25,91,95].

Studies on the olfactory system development in fish species using the plasticity of dendritic spines of GCs of OBs as an index for possible dendritic spines rearrangements are scarce or absent. To date, few descriptive studies exist on the OB’s GCs morphology and prolongations in the common carp *C. carpio* and in the Mediterranean barbel *Barbus meridionalis* (Risso, 1826), where the presence of dendritic spines is reported only in these neurons [85]. Other studies demonstrated the plasticity of these structures in the pyramidal neurons of the optic tectum of the jewel fish *Hemichromis bimaculatus* (Gill, 1862) [100,101,102,103,104,105] and in zebrafish *Danio rerio* (Hamilton, 1882) [106], as well as the spiny-medium-like neurons in the telencephalon of the zebrafish [107].

Therefore, our work represents a pilot study for verifying, first, the presence of the GCs in European eel’s OB, the presence of dendritic spines in this brain area, then the quality evaluation of Golgi-Cox Staining, the morphological characterization of dendritic spines, and their density estimation.

Our findings showed great synaptic development activity in migratory phases of eels, which regresses in the trophic phase, but must necessarily be recreated by tracing the migratory memory of the routes previously travelled in the juvenile phases. If olfaction plays a role in the orientation and migratory behaviour throughout the complex and long life cycle of *A. anguilla* and if eels possess a memory, all these processes could be influenced by changes in the olfactory stimuli under several internal and external conditions (e.g., adaptation to different aquatic environments; sexual development phases, growing or fasting phases, climate changes, ocean alterations) [108,109,110], making it difficult or impossible to cover wide reproductive migratory routes and constituting a cofactor that contributes to threaten this critically endangered species (CR) [111].

For these reasons and due to its ancient phylogenetic attribution to the Elopomorph teleost group, the European eel *A. anguilla* could be recommended as a model species to study and understand the development of olfaction in catadromous fish species [112].

## Figures and Tables

**Table 1:** Biometrics of sampled eels. Mean total length (TL), mean total weight (TW), and brain weight (BW) of sampled eels (± standard deviation) for different life stages.

Life Stage	TL (cm)	TW (g)	BW (g)
Glass eel	6.53 ± 0.43	0.23 ± 0.04	0.004 ± 0.001
Elver	8.06 ± 2.43	0.77 ± 0.94	0.005 ± 0.002
Yellow eel	30.56 ± 3.41	39.04 ± 14.04	0.058 ± 0.026
Silver eel	38.40 ± 3.23	105.75 ± 52.22	0.11 ± 0.049

**Table 2:** Summary of K-W test among eels’ life stages. Paired post hoc Dunn’s test between dendritic spine classes. *p* values < 0.05 are significant. Asterisks indicate the significance code: *p* < 0.0001 = ****; *p* < 0.001 = ***; *p* < 0.01 = **; *p* < 0.05 = *; *p* > 0.05 = ns (non-significant).

	Glass eel		Elver		Yellow eel		Silver eel (M)	
	K-W = 130.19 *p* < 0.001		K-W= 134.86 *p* < 0.001		K-W= 122.46 *p* < 0.001		K-W= 154.93 *p* < 0.001	
**Paired** **groups**	*p* value	significance	*p* value	significance	*p* value	significance	*p* value	significance
**M-S**	<0.0001	****	<0.0001	****	0.4912	Ns	0.0014	**
**M-LT**	0.6921	ns	<0.0001	****	<0.0001	****	<0.0001	****
**M-F**	<0.0001	****	<0.0001	****	<0.0001	****	<0.0001	****
**LT-S**	<0.0001	****	0.3539	ns	0.0028	****	0.051	ns
**LT-F**	<0.0001	****	<0.0001	****	<0.0001	****	<0.0001	****
**S-F**	<0.0001	****	<0.0001	****	<0.0001	****	<0.0001	****

**Table 3:** Summary of K-W test among dendritic spine classes. Paired post hoc Dunn’s test between eels’ life stages. *p* values < 0.05 are significant. Asterisks indicate the significance code: *p* < 0.0001 = ****; *p* < 0.001 = ***; *p* < 0.01 = **; *p* < 0.05 = *; *p* > 0.05 = ns (non-significant).

	Mushroom (M)		Stubby (S)		Long Thin (LT)		Filopodia (F)	
	K-W = 23.52 *p* < 0.001		K-W = 130.19 *p* < 0.001		K-W = 47.23 *p* < 0.001		K-W = 7.90 *p* = 0.048	
**Paired** **groups**	*p* value	significance	*p* value	significance	*p* value	significance	*p* value	significance
**Glass eel-Elver**	0.2479	ns	0.3944	ns	<0.0001	****	0.0793	ns
**Glass eel-Yellow eel**	<0.0001	****	0.5585	ns	<0.0001	****	0.1318	ns
**Glass eel-Silver eel (M)**	0.6584	ns	0.0042	**	<0.0001	****	0.0128	*
**Elver-Yellow eel**	0.0013	**	0.0953	ns	0.7401	ns	0.7591	ns
**Elver-Silver eel (M)**	0.3719	ns	<0.0001	****	0.0145	*	0.3738	ns
**Yellow eel-Silver eel (M)**	<0.0001	****	0.0097	**	0.0040	**	0.2338	ns

## Data Availability

Data are contained within the article.

## References

[1] Kleerekoper H. (1967). Some Aspects of Olfaction in Fishes, with Special Reference to Orientation. Am. Zool..

[2] Hara T.J. (1975). Olfaction in fish. Prog. Neurobiol..

[3] Calvo-Ochoa E., Byrd-Jacobs C.A. (2019). The Olfactory System of Zebrafish as a Model for the Study of Neurotoxicity and Injury: Implications for Neuroplasticity and Disease. Int. J. Mol. Sci..

[4] Gunning G.E. (1959). The sensory basis for homing in the longear sunfish *Lepomis megalotis megalotis* (Rafinesque). Invest. Indiana Lakes Streams.

[5] Creutzberg F. (1961). On the orientation of migrating elvers (*Anguilla vulgaris* Tert.) in a tidal area. Neth. J. Sea Res..

[6] Miles S.G. (1968). Rheotaxis of elvers of the American eel (*Anguilla rostrata*) in the laboratory to water from different streams in Nova Scotia. J. Fish. Res. Bd. Can..

[7] Miles S.G. (1968). Laboratory experiments on the orientation of the adult American eel, *Anguilla rostrata*. J. Fish. Res. Bd. Can..

[8] Thunberg B.E. (1971). Olfaction in parent stream selection by the alewife (*Alosa pseudoharengus*). Anim. Behav..

[9] Atema J., Jacobson S., Todd J., Baylan D. (1973). The importance of chemical signals in stimulating behaviour of marine organisms: Effects of altered environmental chemistry on animal communication. Bioassay Techniques and Environmental Chemistry.

[10] Ramenofsky M., Wingfield J.C. (2007). Regulation of migration. Bioscience.

[11] Oka Y. (1980). The origin of the centrifugal fibers to the olfactory bulb in the goldfish, *Carassius auratus*: An experimental study using the fluorescent dye primuline as a retrograde tracer. Brain Res..

[12] Fujita I., Satou M., Ueda K. (1984). A field-potential study of centripetal and centrifugal connections of the olfactory bulb in the carp, *Cyprinus carpio* (L.). Brain Res..

[13] Murakami T., Morita Y., Ito H. (1983). Extrinsic and intrinsic fiber connections of the telencephalon in a teleost, *Sebastiscus marmoratus*. J. Comp. Neurol..

[14] Rink E., Wullimann M.F. (2004). Connections of the ventral telencephalon (subpallium) in the zebrafish (*Danio rerio*). Brain Res..

[15] Anzai S., Satou M. (1996). Long-term and short-term plasticity in the dendro-dendritic mitral-to-granule cell synapse of the teleost olfactory bulb. Neurosci. Res..

[16] Satou M., Anzai S., Huruno M. (2005). Long-term potentiation and olfactory memory formation in the carp (*Cyprinus carpio* L.) olfactory bulb. J. Comp. Physiol. A.

[17] Satou M., Hoshikawa R., Sato Y., Okawa K. (2006). An in vitro study of long-term potentiation in the carp (*Cyprinus carpio* L.) olfactory bulb. J. Comp. Physiol. A.

[18] Rehn B., Breipohl W., Mendoza A.S., Apfelbach R. (1986). Changes in granule cells of the ferret olfactory bulb associated with imprinting on prey odours. Brain Res..

[19] Matsutani S., Yamamoto N. (2004). Postnatal development of dendritic spines on olfactory bulb granule cells in rats. J. Comp. Neurol..

[20] Matsutani S., Yamamoto N. (2004). Brain-derived neurotrophic factor induces rapid morphological changes in dendritic spines of olfactory bulb granule cells in cultured slices through the modulation of glutamatergic signaling. Neuroscience.

[21] Mizrahi A. (2007). Dendritic development and plasticity of adult-born neurons in the mouse olfactory bulb. Nat. Neurosci..

[22] Huang Y.B., Hu C.R., Zhang L., Yin W., Hu B. (2015). In vivo study of dynamics and stability of dendritic spines on olfactory bulb interneurons in *Xenopus laevis* tadpoles. PLoS ONE.

[23] McDole B., Isgor C., Pare C., Guthrie K. (2015). BDNF over-expression increases olfactory bulb granule cell dendritic spine density in vivo. Neuroscience.

[24] Mandairon N., Kuczewski N., Kermen F., Forest J., Midroit M., Richard M., Thevenet M., Sacquet J., Linster C., Didier A. (2018). Opposite regulation of inhibition by adult-born granule cells during implicit versus explicit olfactory learning. eLife.

[25] Zhang L., Huang Y., Hu B. (2016). Olfactory experiences dynamically regulate plasticity of dendritic spines in granule cells of *Xenopus* tadpoles in vivo. Sci. Rep..

[26] Alvarez V.A., Sabatini B.L. (2007). Anatomical and physiological plasticity of dendritic spines. Annu. Rev. Neurosci..

[27] Matsuzaki M., Ellis-Davies G.C., Nemoto T., Miyashita Y., Iino M., Kasai H. (2001). Dendritic spine geometry is critical for AMPA receptor expression in hippocampal CA1 pyramidal neurons. Nat. Neurosci..

[28] Matsuzaki M., Honkura N., Ellis-Davies G.C., Kasai H. (2004). Structural basis of long-term potentiation in single dendritic spines. Nature.

[29] Ghani M.U., Mesadi F., Kanık S.D., Argunşah A.Ö., Hobbiss A.F., Israely I., Ünay D., Taşdizen T. (2017). Çetin, M. Dendritic spine classification using shape and appearance features based on two-photon microscopy. J. Neurosci. Methods.

[30] Spiga S., Talani G., Mulas G., Licheri V., Fois G.R., Muggironi G., Masala N., Cannizzaro C., Biggio G., Sanna E. (2014). Hampered long-term depression and thin spine loss in the nucleus accumbens of ethanol-dependent rats. Biol. Sci..

[31] Pchitskaya E., Bezprozvanny I. (2020). Dendritic spines shape analysis—Classification or clusterization? Perspective. Front. Synaptic Neurosci..

[32] Hayashi Y., Majewska A.K. (2005). Dendritic spine geometry: Functional implication and regulation. Neuron.

[33] Bourne J., Harris K.M. (2007). Do thin spines learn to be mushroom spines that remember?. Curr. Opin. Neurobiol..

[34] Hering H., Sheng M. (2001). Dentritic spines: Structure, dynamics and regulation. Nat. Rev. Neurosci..

[35] Yoshihara Y., De Roo M., Muller D. (2009). Dendritic spine formation and stabilization. Curr. Opin. Neurobiol..

[36] Berry K.P., Nedivi E. (2017). Spine dynamics: Are they all the same?. Neuron.

[37] Kasai H., Fukuda M., Watanabe S., Hayashi-Takagi A., Noguchi J. (2010). Structural dynamics of dendritic spines in memory and cognition. Trends Neurosci..

[38] Lendvai B., Stern E.A., Chen B., Svoboda K. (2000). Experience-dependent plasticity of dendritic spines in the developing rat barrel cortex in vivo. Nature.

[39] Muller D., Nikonenko I., Rubenstein J., Rakic P. (2013). Dendritic spines. Neural Circuit Development and Function in the Healthy and Diseased Brain. Comprehensive Developmental Neuroscience.

[40] Fu M., Zuo Y. (2011). Experience-dependent structural plasticity in the cortex. Trends Neurosci..

[41] Yang G., Pan F., Gan W.B. (2009). Stably maintained dendritic spines are associated with lifelong memories. Nature.

[42] Zhou Q., Homma K.J., Poo M.M. (2004). Shrinkage of dendritic spines associated with long-term depression of hippocampal synapses. Neuron.

[43] Bailey C.H., Kandel E.R., Harris K.M. (2015). Structural components of synaptic plasticity and memory consolidation. Cold Spring Harb. Perspect. Biol..

[44] Tesch F.W. (2003). The Eel.

[45] Schmidt J. (1923). Breeding Places and Migrations of the Eel. Nature.

[46] Miller M.J., Westerberg H., Sparholt H., Wysujack K., Sørensen S.R., Marohn L., Jacobsen M.W., Freese M., Ayala D.J., Pohlmann J.D. (2019). Spawning by the European eel across 2000 km of the Sargasso Sea. Biol. Lett..

[47] Tesch F.W. (1977). The Eel Biology and Management of Anguillid Eels.

[48] Bonhommeau S., Castonguay M., Rivot E., Sabatié R., Le Pape O. (2010). The duration of migration of Atlantic Anguilla larvae. Fish Fish..

[49] Hanel R., Stepputtis D., Bonhommeau S., Castonguay M., Schaber M., Wysujack K., Vobach M., Miller M.J. (2014). Low larval abundance in the Sargasso Sea: New evidence about reduced recruitment of the Atlantic eels. Naturwissenschaften.

[50] Miller M.J., Bonhommeau S., Munk P., Castonguay M., Hanel R., McCleave J.D. (2015). A century of research on the larval distributions of the Atlantic eels: A re-examination of the data. Biol. Rev..

[51] Tesch F.W. (1980). Occurrence of Eel *Anguilla anguilla* Larvae West of the European Continental Shelf, 1971–1977. Environ. Biol. Fishes.

[52] Deelder C.L. (1952). On the migration of the elver (*Anguilla vulgaris* Turt.) at sea. J. Cons. Int. Explor. Mer..

[53] Tosi L., Sala L., Sola C., Spampanato A., Tongiorgi P. (1988). Experimental analysis of the thermal and salinity preferences of glass-eels, *Anguilla anguilla* (L.), before and during the upstream migration. Fish Biol..

[54] Crnjar R., Slcalera G., Bigiani A., Tomassini Barbarossa I., Magherini P.C., Pietra P. (1992). Olfactory sensitivity to amino acids in the juvenile stages of the European eel *Anguilla anguilla* (L.). J. Fish Biol..

[55] Sola C., Tosi L. (1993). Bile salts and taurine as chemical stimuli for glass eels, *Anguilla anguilla*: A behavioural study. Environ. Biol. Fishes.

[56] Sola C. (1995). Chemoattraction of upstream migrating glass eels *Anguilla anguilla* to earthy and green odorants. Environ. Biol. Fishes.

[57] Schmucker A.K., Johnson N.S., Galbraith H.S., Li W. (2016). Glass-eel-stage American eels respond to conspecific odor as a function of concentration. Trans. Am. Fish. Soc..

[58] Cresci A., Paris C.B., Durif C.M., Shema S., Bjelland R.M., Skiftesvik A.B., Browman H.I. (2017). Glass eels (*Anguilla anguilla*) have a magnetic compass linked to the tidal cycle. Sci. Adv..

[59] Cresci A., Paris C.B., Foretich M.A., Durif C.M., Shema S.D., O’Brien C.E., Vikebø F.B., Skiftesvik A.B., Browman H.I. (2019). Atlantic haddock (*Melanogrammus aeglefinus*) larvae have a magnetic compass that guides their orientation. iScience.

[60] Cresci A., Durif C.M., Paris C.B., Shema S.D., Skiftesvik A.B., Browman H.I. (2019). Glass eels (*Anguilla anguilla*) imprint the magnetic direction of tidal currents from their juvenile estuaries. Commun. Biol..

[61] Tzeng W.N., Wang C.H., Wickström M.H., Reizenstein M. (2000). Occurrence of the semi-catadromous European eel *Anguilla anguilla* in the Baltic Sea. Mar. Biol..

[62] Wood P., Partridge J.C., Grip W.J. (1992). Rod visual pigment changes in the elver of the eel *Anguilla anguilla* L. measured by microspectrophotometry. J. Fish Biol..

[63] Ciccotti B.E., Macchi E., Rossi A., Cataldi E., Cataudella S. (1993). Glass eel (*Anguilla anguilla*) acclimation to freshwater and seawater: Morphological changes of the digestive tract. J. Appl. Ichthyol..

[64] Durif C.M.F., Van Ginneken V., Dufour S., Müller T., Elie P. (2009). Seasonal evolution and individual differences in silvering eels from different locations. Spawning Migration of the European Eel.

[65] Amilhat E., Aarestrup K., Faliex E., Simon G., Westerberg H., Righton D. (2016). First evidence of European eels exiting the Mediterranean Sea during their spawning migration. Sci. Rep..

[66] Righton D., Westerberg H., Feunteun E., Okland F., Gargan P., Amilhat E., Metcalfe J., Lobon-Cervia J., Sjo Berg N., Simon J. (2016). Empirical observations of the spawning migration of European eels: The long and dangerous road to the Sargasso Sea. Sci. Adv..

[67] Béguer-Pon M., Dodson J.J., Castonguay M., Jellyman D., Aarestrup K., Tsukamoto K. (2018). Tracking anguillid eels: Five decades of telemetry-based research. Mar. Freshw. Res..

[68] Huertas M., Canário A.V.M., Hubbard P.C. (2008). Chemical Communication in the Genus *Anguilla*: A Minireview. Behaviour.

[69] Westin L. (1990). Orientation mechanisms in migrating European silver eel (*Anguilla anguilla*): Temperature and olfaction. Mar. Biol..

[70] Westin L. (1998). The spawning migration of European silver eel (*Anguilla anguilla* L.) with particular reference to stocked eel in the Baltic. Fish. Res..

[71] Westin L. (2003). Migration failure in stocked eels *Anguilla anguilla*. Mar. Ecol. Prog. Ser..

[72] De Waele J., Martina M.L.V., Sanna L., Cabras S., Cossu Q.A. (2010). Flash flood hydrology in karstic terrain: Flumineddu Canyon, 446 central-east Sardinia. Geomorphology.

[73] Sabatini A., Podda C., Frau G., Cani M.V., Musu A., Serra M., Palmas F. (2018). Restoration of native Mediterranean brown trout *Salmo cettii* Rafinesque, 1810 (Actinopterygii: Salmonidae) populations using an electric barrier as a mitigation tool. Eur. Zool. J..

[74] Palmas F., Righi T., Musu A., Frongia C., Podda C., Serra M., Splendiani A., Caputo Barucchi V., Sabatini A. (2020). Pug-headed-ness anomaly in a wild and isolated population of native mediterranean trout *Salmo trutta* L.; 1758 complex (Osteichthyes: Salmonidae). Dyversity.

[75] Podda C., Palmas F., Pusceddu A., Sabatini A. (2022). When the eel meets dams: Larger dams’ long-term impacts on *Anguilla anguilla* (L., 1758). Front. Environ. Sci..

[76] Regione Autonoma della Sardegna (2022). Carta Ittica Della Sardegna—D.G.R. N. 2/28, 428. del 20/01/2022.

[77] Podda C., Palmas F., Frau G., Chessa G., Culurgioni J., Diciotti R., Fois N., Sabatini A. (2020). Environmental influences on the ecruitment dynamics of juvenile European eels, *Anguilla anguilla*, in a small estuary of the Tyrrhenian Sea, Sardinia, Italy. Aquat. Conserv. Mar. Freshw. Ecosyst..

[78] Gilderhus P.A., Marking L.L. (1987). Comparative efficacy of 16 anesthetic chemicals on rainbow trout. N. Am. J. Fish. Manag..

[79] Cox W.H. (1891). Imprägnation des centralen Nervensystemsmit Quecksilbersalzen. Arch. Mikrosk. Anat..

[80] Glaser E.M., Van der Loos H. (1981). Analysis of thick brain sections by obverse—Reverse computer microscopy: Application of a new, high clarity Golgi—Nissl stain. J. Neurosci. Methods.

[81] Zaqout S., Kaindl A.M. (2016). Golgi-Cox staining step by step. Front. Neuroanat..

[82] Wullimann M., Rupp B., Reichert H. (1996). Neuroanatomy of the Zebrafish Brain. A Topological Atlas.

[83] Kolb B., McClimans J. (1986). Cryostat sectioning of Golgi-Cox tissue. Stain Technol..

[84] Medina M., Reperant J., Dufour S., Ward R., Le Belle N., Miceli D. (1994). The distribution of GABA-immunoreactive neurons in the brain of the silver eel (*Anguilla anguilla* L.). Anat. Embryol..

[85] Alonso J.R., Lara J., Miguel J.J., ON J. (1986). A Golgi study of the granule cells in the olfactory bulb of *Cyprinus carpio* L. and *Barbus meridionalis* Risso. Z. Mikrosk.-Anat. Forsch..

[86] R Core Team (2021). R: A Language and Environment for Statistical Computing.

[87] Døving K.B., Autrum H., Ottoson D., Perl E.R., Schmidt R.F., Shimazu H., Willis W.D. (1986). Functional Properties of the Fish Olfactory System. Progress in Sensory Physiology 6. Progress in Sensory Physiology.

[88] Okabe S., Kim H.D., Miwa A., Kuriu T., Okado H. (1999). Continual remodeling of postsynaptic density and its regulation by synaptic activity. Nat. Neurosci..

[89] Dickstein D.L., Weaver C.M., Luebke J.I., Hof P.R. (2013). Dendritic spine changes associated with normal aging. Neuroscience.

[90] Peters A., Kaiserman-Abramof I.R. (1970). The small pyramidal neuron of the rat cerebral cortex. The perikaryon, dendrites and spines. Am. J. Anat..

[91] Harris K.M., Jensen F.E., Tsao B. (1992). Three-dimensional structure of dendritic spines and synapses in rat hippocampus (CA1) at postnatal day 15 and adult ages: Implications for the maturation of synaptic physiology and long-term potentiation. J. Neurosci..

[92] Stewart M.G., Medvedev N.I., Popov V.I., Schoepfer R., Davies H.A., Murphy K., Dallérac G.M., Kraev I.V., Rodrìguez J.J. (2005). Chemically induced long-term potentiation increases the number of perforated and complex postsynaptic densities but does not alter dendritic spine volume in CA1 of adult mouse hippocampal slices. Eur. J. Neurosci..

[93] Harris K.M. (1999). Structure, development, and plasticity of dendritic spines. Curr. Opin. Neurobiol..

[94] Fiala J.C., Allwardt B., Harris K.M. (2002). Dendritic spines do not split during hippocampal LTP or maturation. Nat. Neurosci..

[95] Brocco M.A., Fernández M.E., Frasch A.C. (2010). Filopodial protrusions induced by glycoprotein M6a exhibit high motility and aids synapse formation. Eur. J. Neurosci..

[96] Fiala J.C., Feinberg M., Popov V., Harris K.M. (1998). Synaptogenesis via dendritic filopodia in developing hippocampal area CA1. J. Neurosci..

[97] Ziv N.E., Smith S.J. (1996). Evidence for a role of dendritic filopodia in synaptogenesis and spine formation. Neuron.

[98] Sorra K.E., Harris K.M. (2000). Overview on the structure, composition, function, development, and plasticity of hippocampal dendritic spines. Hippocampus.

[99] Churcher A.M., Hubbard P.C., Marques J.P., Canário A.V., Huertas M. (2015). Deep sequencing of the olfactory epithelium reveals specific chemosensory receptors are expressed at sexual maturity in the European eel *Anguilla anguilla*. Mol. Ecol..

[100] Coss R.G. (1979). Delayed plasticity of an instinct: Recognition and avoidance of 2 facing eyes by the jewel fish. Dev. Psychobiol..

[101] Coss R.G., Globus A. (1978). Spine stems on tectal interneurons in jewel fish are shortened by social stimulation. Science.

[102] Coss R.G., Globus A. (1979). Social experience affects the development of dendritic spines and branches on tectal interneurons in the jewel fish. Dev. Psychobiol..

[103] Coss R.G., Burgess J.W. (1981). Jewel fish retain juvenile schooling pattern after crowded development. Dev. Psychobiol..

[104] Berard D.R., Burgess J.W., Coss R.G. (1981). Plasticity of dendritic spine formation: A state-dependent stochastic process. Int. J. Neurosci..

[105] Burgess J.W., Coss R.G. (1980). Crowded jewel fish show changes in dendritic spine density and spine morphology. Neurosci. Lett..

[106] Plata A.L.D., Robles E. (2022). NMDA Receptor Antagonist MK801 Reduces Dendritic Spine Density and Stability in Zebrafish Pyramidal Neurons. Neuroscience.

[107] Song C., Liu B.P., Zhang Y.P., Peng Z., Wang J., Collier A.D., Echevarria D.J., Savelieva K.V., Lawrence R.F., Rex C.S. (2018). Modeling consequences of prolonged strong unpredictable stress in zebrafish: Complex effects on behavior and physiology. Prog. Neuropsychopharmacol. Biol. Psychiatry..

[108] Bevacqua D., Melià P., Gatto M., De Leo G.A. (2015). A global viability assessment of the European eel. Glob. Change Biol..

[109] Podda C., Palmas F., Pusceddu A., Sabatini A. (2021). Hard times for catadromous fish: The case of the European eel *Anguilla anguilla* (L. 1758). Adv. Oceanogr. Limnol..

[110] Durif C.M.F., Gjosaeter J., Vollestad L.A. (2011). Influence of oceanic factors on *Anguilla anguilla* (L.) over the twentieth century in coastal habitats of the Skagerrak, Southern Norway. Proc. R. Soc. B.

[111] Pike C., Crook V., Gollock M. (2020). *Anguilla anguilla*. The IUCN Red List of Threatened Species.

[112] Lauder G.V., Liem K.F., Northcutt R.G., Davis R.E. (1983). Patterns of diversity and evolution in ray-finned fishes. Fish Neurobiology. Brain Stem and Sense Organs.

